# Global Burden of Disease 2023: Challenges and opportunities for a growing collaboration

**DOI:** 10.1371/journal.pmed.1004838

**Published:** 2025-11-26

**Authors:** Zulfiqar A. Bhutta

**Affiliations:** 1 Institute for Global Health & Development, The Aga Khan University, South-Central Asia, East Africa & United Kingdom; 2 SickKids Centre for Global Child Health, Hospital for Sick Children, Toronto, Canada; 3 Department of Paediatrics, Nutritional Sciences and Public Health, University of Toronto, Toronto, Canada

## Abstract

In this Editorial, Zulfiqar Bhutta discusses the Global Burden of Disease (GBD) 2023 report, outlining why, alongside improved health monitoring, data acquisition, and analytical methods, its continued expansion creates new challenges and opportunities for improving its accuracy, completeness, external validity, and policy relevance.

## Teaser

The Global Burden of Disease 2023 represents the most comprehensive iteration of its kind since first reported in 1993. Despite improved health monitoring, data acquisition, and analytical methods, its expansion creates new challenges and opportunities for improving its accuracy, completeness, external validity, and policy relevance.

The Global Burden of Disease (GBD) study has now been established for over three decades [1]. The study began in 1991 and when published in 1993, estimated the burden of disease for 106 conditions and 10 risk factors across eight world regions, disaggregated into five age groups for the year 1990. The GBD developed the unique concept of disability adjusted life years (DALYs) to provide a composite measure combining years of life lost due to premature death and healthy years of life lost due to disability [2]. Hosted at the Institute for Health Metrics (IHME) since 2010, the GBD has become a source of annual updates with estimates of disease burden, risk factors, and trends across 204 countries and 660 subnational locations.

Successive GBD editions have relied on a burgeoning network of collaborators (now exceeding 17,000 from 167 countries), who alongside a core group of experts at the IHME, constantly improve data sources and analytical methods to produce and update global estimates every two years. These methods have increasingly benefited from a growing range of data sources, including vital registration systems as well as large national and subnational datasets and registries. Remote sensing methodologies and geospatial localization of the data has also improved precision, although accurate and timely information from many low- and middle-income countries (LMICs) remains a barrier.

The most recent GBD2023 results were launched in Berlin at the World Health Summit 2025, accompanied by capstone papers on mortality and life expectancy [3], causes of death [4], and quantification of 88 risk factors and healthy life expectancy [5]. Several new findings emerge from the GBD2023 analysis. Firstly, that prior estimates of mortality in Africa could have over-estimated mortality rates and DALYs among those over 50 years of age. Contrastingly, the study presents a lower life expectancy among those under 20 years of age in the Sahel region of Africa. The data also show that despite the excess mortality from the COVID-19 period, most countries had recovered from the pandemic by 2023. GBD2023 also underscored the enormous increase in non-communicable diseases (such as cardiovascular disease, diabetes, and stroke) across the world, especially in middle-income countries. For the major GBD risk factors (behavioral, metabolic, environmental, and occupational risks), the attributable DALYs have risen by 30.7% since 2010 [5]. GBD2023 also underscored the significant increases in levels of obesity, as well as the burden of mental health disorders, and highlighted that these were made worse by social determinants and systemic challenges. GBD2023 also highlighted the steadily growing contribution of high environmental temperature to excess mortality and DALYs in low- and lower-middle-income countries, alongside other environmental risk factors such as air pollution and exposure to lead.

Methodologically, the GBD2023 report is notable in several aspects. Compared to previous iterations, there were major improvements in its comprehensiveness, with a greater number of data sources (24,025 compared to 22,223 in GBD 2021), complete birth histories for the 5–14 years age group, age-specific sibling histories for those between 15 and 49 years of age, and a single integrated statistical model (OneMod) incorporating both parametric and non-parametric methods.

These advances notwithstanding, there are still several limitations with the GBD2023 estimates. Some of these are recognized through the lack of granular and sub-national information from routine information systems in many LMICs, given the dependency on national level cross-sectional sample surveys. At times, given the inevitable limits of modeling, this can lead to counterintuitive findings, such as the reported changes in annual mortality rates between 2019 and 2023 in many African and Asian countries, especially where there are no annual surveys reporting on mortality therein [3]. It is also notable that some of the countries in polycrises (e.g., conflict, climate change and migration), with the probable exception of the Sahel region, have the least amount of information at the population level. Hence, some of the observed differences in risk-attributable DALYs could reflect a paucity of information and uncertainties of modeling. To illustrate, the risk-attributable child growth failure DALYs in Yemen and Sudan in the recent estimates [3] are several folds less than neighboring Somalia and Ethiopia, which does not match global childhood undernutrition estimates [6].

There is also a clear need to align GBD2023 with other global initiatives in terms of terminology and methods. While GBD2023 presents risk maps related to low birth weight and short gestation, it would be more programmatically useful to present the data as categories of small vulnerable newborn births [7], given the need to align interventions for prevention and management.

There are also huge opportunities for global health metrics as countries take charge of their own information systems, an issue already underscored by investigators from LMICs [8]. A priority is investment in vital registration systems and improvements in sentinel reporting systems [9]. Such investments also reflect national priorities, regardless of economic status or resources. For example, Kyrgyz Republic, a country with a GDP per capita income around US$ 1,200 per annum, could implement and maintain a complete national birth registry, allowing for granular subnational analysis of progress and disparities [10].

The district health information system, an open-access platform for collecting and analyzing aggregated data from public sector facilities [11], is now in place in more than 80 countries. Opportunities abound for linking other routine data collection systems with such DHIS data and concomitantly strengthening health reporting systems. To illustrate, Pakistan [12] and Afghanistan [13] have long had both systems in place yet largely rely on cross-sectional surveys with little emphasis on building routine information systems. With improvements in big data analytics, remote sensing, geospatial methods, and newer methods to assess population clusters and settlements [14], there are additional opportunities for further refinement of GBD estimates at the subnational level.

Finally, the GBD has long used the composite socio-demographic index (SDI), based on lag-distributed income per capita, average years of schooling for individuals aged 15 and older, and total fertility rate among women younger than 25 years. The SDI is used for assessing background social and economic contexts of relevance to health outcomes and characterizing differentials, but may not capture many of the additional contextual factors that help shape health outcomes, such as climate change, other environmental risks, conflict, health system functionality, etc. It may therefore be worthwhile exploring some of these contextual measures as well to increase the policy relevance of GBD2023 estimates.

The value of GBD and related metrics as a major global innovation and public goods cannot be emphasized enough. As the scope and granularity of the GBD estimates and network of collaborators increase with each successive iteration, closer attention should be paid to data quality and completeness and country-level ownership. With the cessation of US support for demographic and health surveys, this also presents a wealth of opportunities for methodological improvements, democratization, and transparency in health information systems.

## Abbreviations

DALYs: disability adjusted life years
GBD: Global Burden of Disease
IHME: Institute for Health Metrics
LMICs: low- and middle-income countries
SDI: socio-demographic index

## References

[1] Murray CJL. The Global Burden of Disease Study at 30 years. Nat Med. 2022;28(10):2019–26. doi: 10.1038/s41591-022-01990-1 36216939

[2] Murray CJ, Lopez AD, Jamison DT. The global burden of disease in 1990: summary results, sensitivity analysis and future directions. Bull World Health Organ. 1994;72(3):495–509. 8062404 PMC2486716

[3] GBD 2023 Demographics Collaborators. Global age-sex-specific all-cause mortality and life expectancy estimates for 204 countries and territories and 660 subnational locations, 1950-2023: a demographic analysis for the Global Burden of Disease Study 2023. Lancet. 2025;406(10513):1731–810. doi: 10.1016/S0140-6736(25)01330-3 41092927 PMC12535839

[4] GBD 2023 Causes of Death Collaborators. Global burden of 292 causes of death in 204 countries and territories and 660 subnational locations, 1990-2023: a systematic analysis for the Global Burden of Disease Study 2023. Lancet. 2025;406(10513):1811–72. doi: 10.1016/S0140-6736(25)01917-8 41092928 PMC12535838

[5] GBD 2023 Disease and Injury and Risk Factor Collaborators. Burden of 375 diseases and injuries, risk-attributable burden of 88 risk factors, and healthy life expectancy in 204 countries and territories, including 660 subnational locations, 1990-2023: a systematic analysis for the Global Burden of Disease Study 2023. Lancet. 2025;406(10513):1873–922. doi: 10.1016/S0140-6736(25)01637-X 41092926 PMC12535840

[6] UNICEF-WHO-The World Bank: Joint Child Malnutrition Estimates (JME)—levels and trends—2025 edition; 2025. Available from: https://data.unicef.org/resources/jme/

[7] Ashorn P, Ashorn U, Muthiani Y, Aboubaker S, Askari S, Bahl R, et al. Small vulnerable newborns-big potential for impact. Lancet. 2023;401(10389):1692–706. doi: 10.1016/S0140-6736(23)00354-9 37167991

[8] Kiwuwa-Muyingo S, Kengne AP. USAID defunding: an investment call for low-income and middle-income countries. Lancet. 2025;406(10500):209–11. 40683700 10.1016/S0140-6736(25)01398-4

[9] Grover A, Nair S, Sharma S, Gupta S, Shrivastava S, Singh P, et al. strengthening cause of death statistics in selected districts of 3 states in India: protocol for an uncontrolled, before-after, mixed method study. JMIR Res Protoc. 2024;13:e51493. doi: 10.2196/51493 39705697 PMC11699485

[10] Kamali M, Wright JE, Akseer N, Tasic H, Conway K, Brar S, et al. Trends and determinants of newborn mortality in Kyrgyzstan: a Countdown country case study. Lancet Glob Health. 2021;9(3):e352–60. doi: 10.1016/S2214-109X(20)30460-5 33308422 PMC7886658

[11] DHIS2 and HISP: supporting local information systems worldwide. https://dhis2.org/

[12] GBD 2019 Pakistan Collaborators. The state of health in Pakistan and its provinces and territories, 1990-2019: a systematic analysis for the Global Burden of Disease Study 2019. Lancet Glob Health. 2023;11(2):e229–43. 10.1016/S2214-109X(22)00497-1 PMC10009760 36669807

[13] Alba S, Sondorp E, Kleipool E, Yadav RS, Rahim AS, Juszkiewicz KT, et al. Estimating maternal mortality: what have we learned from 16 years of surveys in Afghanistan?. BMJ Glob Health. 2020;5(5):e002126. doi: 10.1136/bmjgh-2019-002126 32371572 PMC7228470

[14] Darooneh AH, Kortenaar J-L, Goulart CM, McLaughlin K, Cornelius SP, Bassani DG. SEEDNet: Covariate-free multi-country settlement-level epidemiological estimates datasets for network analysis. Sci Data. 2025;12(1):975. doi: 10.1038/s41597-025-05143-0 40494856 PMC12152140

